# Compressed cerebro‐cerebellar functional gradients in children and adolescents with attention‐deficit/hyperactivity disorder

**DOI:** 10.1002/hbm.26796

**Published:** 2024-09-10

**Authors:** Qingquan Cao, Pan Wang, Ziqian Zhang, F. Xavier Castellanos, Bharat B. Biswal

**Affiliations:** ^1^ The Clinical Hospital of Chengdu Brain Science Institute, MOE Key Laboratory for Neuroinformation, Center for Information in Medicine, School of Life Science and Technology University of Electronic Science and Technology of China Chengdu China; ^2^ Department of Child and Adolescent Psychiatry New York University Grossman School of Medicine New York New York USA; ^3^ Nathan Kline Institute for Psychiatric Research Orangeburg New York USA; ^4^ Department of Biomedical Engineering New Jersey Institute of Technology Newark New Jersey USA

**Keywords:** ADHD, age, cerebro‐cerebellar functional gradient, compression pattern, interaction effect

## Abstract

Both cortical and cerebellar developmental differences have been implicated in attention‐deficit/hyperactivity disorder (ADHD). Recently accumulating neuroimaging studies have highlighted hierarchies as a fundamental principle of brain organization, suggesting the importance of assessing hierarchy abnormalities in ADHD. A novel gradient‐based resting‐state functional connectivity analysis was applied to investigate the cerebro‐cerebellar disturbed hierarchy in children and adolescents with ADHD. We found that the interaction of functional gradient between diagnosis and age was concentrated in default mode network (DMN) and visual network (VN). At the same time, we also found that the opposite gradient changes of DMN and VN caused the compression of the cortical main gradient in ADHD patients, implicating the co‐occurrence of both low‐ (visual processing) and high‐order (self‐related thought) cognitive dysfunction manifesting in abnormal cerebro‐cerebellar organizational hierarchy in ADHD. Our study provides a neurobiological framework to better understand the co‐occurrence and interaction of both low‐level and high‐level functional abnormalities in the cortex and cerebellum in ADHD.

## Graphical abstract

We found the abnormal interaction of diagnosis and age in DMN (default mode network) and VN (visual network) in attention‐deficit/hyperactivity disorder (ADHD). The opposite gradient change of VN portion and DMN portion leads to the compression of cortex in ADHD.

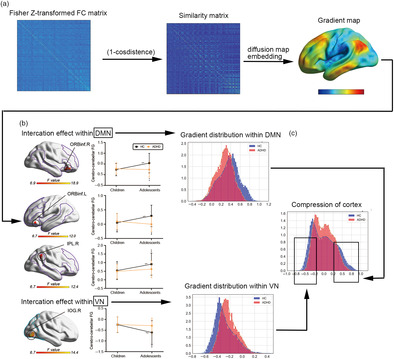

## 1 INTRODUCTION

Attention‐deficit/hyperactivity disorder (ADHD), the most common childhood neurodevelopmental disorder, is characterized clinically by inattention, impulsivity, and hyperactivity and frequently by learning difficulties (Polanczyk et al., 2007). ADHD symptoms often endure into adulthood and can co‐occur with oppositional defiant disorder, anxiety disorders, conduct disorder, and tic disorders, with serious negative effects on family and society, including increased risks of suicide and criminal activity (Shi et al., 2021). Accumulating neuroimaging studies have implicated structural and functional abnormalities involving the cerebellum and cortex in ADHD (Chen et al., 2022; Duan et al., 2023; Hoogman et al., 2017; Yu et al., 2016). Most previous studies focused on the abnormalities in the local cortex or cerebellum of ADHD patients, while ignoring the co‐occurrence and interplay of low‐ and high‐level functional abnormalities. Therefore, a neurobiological basis to explore the abnormalities in the cortex and cerebellum of ADHD patients from a global perspective remains to be established.

In addition to modulating motor coordination, the cerebellum also modulates high‐order cognitive functions, including expression of emotions, attention control, and response timing (Jacobi et al., 2021; Yuan et al., 2022). Neurodevelopmental disorders, including autism spectrum disorder, intellectual disability, and ADHD, have revealed anatomic and functional disruption in the cerebellum (Sathyanesan et al., 2019). Cerebellar findings in ADHD include cerebellar vermis atrophy related to inattentive and hyperactive indices (Chang et al., 2021), increased functional connectivity within the cerebellum (Zhao et al., 2022), and decreased cerebellar activation during a verbal working memory paradigm (Yap et al., 2021). In a longitudinal study, Castellanos and colleagues found a lower developmental trajectory for cerebellar volume in children with ADHD between the ages of 5–18 years compared with healthy controls (HC; Castellanos et al., 2002). On a cognitive control task, O'Halloran et al. (2018) reported increased functional connectivity between the prefrontal cortex and cerebellum in children with ADHD ages 5–17 years. We previously reported increased functional connectivity between the superior temporal gyrus and cerebellum in ADHD, which also increased in relation to age (Wang et al., 2022). Thus, multi‐modal neuroimaging in ADHD has implicated the cerebellum across developmental stages.

The effects of ADHD must be considered in the context of development. Compared with children, adolescents with ADHD are more likely to develop anxiety disorders and have conflicts with parents and abuse substances (Biederman et al., 1998). As age increases, ADHD patients tend to smoke, drink, use illegal drugs (Marshal & Molina, 2006), and are more likely to become homeless (García et al., 2016). While differences in clinical characteristics have been reported extensively between children and adolescents with ADHD (Evans et al., 2014; Goldman et al., 1998), the corresponding neuroimaging correlates remain unclear. Specifically, cerebro‐cerebellar hierarchical relationships have not been investigated in ADHD.

The hierarchical structure of neural information in the human brain can be described in terms of multimodal and multi‐scale representations, that is, gradients (Raut et al., 2020). The starting point for quantifying the hierarchy of cortex or cerebellum is to divide the cortex or cerebellum into a number of discrete communities with clear boundaries (Buckner et al., 2011; Damoiseaux et al., 2006; Yeo et al., 2011). Unlike prior commonly used methods, gradient‐based analyses use diffusion graph embedding to perform nonlinear decompositions of the high‐dimensional resting‐state functional connectivity matrix (Coifman et al., 2005; Margulies et al., 2016), generating low‐dimensional embeddings. The resulting diffusion embedding is not a single mosaic of a discrete network, but multiple continuous maps (namely gradients) capturing the voxel‐level similarity of functional connectivity along a continuous space. Within a given connectivity gradient, voxels with similar functional connectivity patterns are close to each other (Huntenburg et al., 2018). Therefore, gradient‐based analysis methods describe different functional modules in a continuous manner, while reflecting the hierarchical organization of brain function (Bayrak et al., 2019; Mesulam, 2012). To consolidate the co‐occurrence and interplay of low‐ and high‐level functional abnormalities, researchers have proposed the gradient‐based analysis method that could provide a framework to explore the functional hierarchy changes in the cortex and cerebellum from a global perspective.

This study aimed to investigate the abnormality of cerebro‐cerebellar functional gradient (FG) in children and adolescents with ADHD. We hypothesize that individuals with ADHD exhibit the interaction effects of diagnosis and age distributed in the cortex and cerebellum, and the disorder of functional hierarchy is caused by the co‐occurrence and interplay of low‐ and high‐level functional abnormalities. To the aim, we applied a novel gradient‐based resting‐state functional connectivity analysis to investigate the interactions between diagnosis (ADHD vs. HC) and age (Children vs. Adolescents) in cerebro‐cerebellar FG. Then a histogram analysis was performed on the cerebro‐cerebellar FG maps to investigate distributional differences in ADHD. This study promoted the understanding of developmental process relating to cerebro‐cerebellar disturbed hierarchy in ADHD.

## 2 METHODS

### 2.1 Participants

This study used the publicly available ADHD‐200 dataset from two sites, namely the New York University (NYU) Child Study Center and Peking University (PKU) (http://fcon_1000.projects.nitrc.org/indi/adhd200/). ADHD diagnoses were based on the Attention‐Deficit/Hyperactivity Disorder Rating Scale‐IV (ADHD‐RS‐IV) and the Conners' Parent Rating Scale‐Revised, Long version (CPRS‐R‐LV) at PKU and NYU, respectively (Hong & Hwang, 2022). The datasets from NYU and PKU included 152 ADHD/111 HC, and 102 ADHD/143 HC, respectively. We excluded 208 participants (110 ADHD and 98 HC) for the following reasons: (1) Missing resting‐state functional images (5 ADHD and 1 HC); (2) Low image quality including incomplete images and marked artifacts (29 ADHD and 26 HC); (3) Maximum head motion exceeding 2.0 mm or 2.0 degrees (37 ADHD and 31 HC); (4) Missing phenotypic information such as sex or clinical information (22 ADHD and 17 HC); (5) Finally, to obviate significant between‐group differences in sex, we randomly excluded 17 ADHD and 23 HC.

Previous research has found that functional connectivity in higher cognitive networks (DMN and frontoparietal network [FPN]) peaks at age 12 (DeSerisy et al., 2021). American Academy of Child and Adolescent Psychiatry divided patients into elementary‐school‐age children (6–11 years), and adolescents (12–18 years) at the age of 12 to configure different management recommendation (ADHD, 2011). This study divided ADHD patients into children and adolescents at the age of 12 to investigate the interactions between diagnosis (ADHD vs. HC) and age (Children vs. Adolescents) in cerebro‐cerebellar FG in line with our previous study (Wang et al., 2022). Subjects were divided into two subgroups comprising children (age from 7.0 up to [but not including] 12.0) and adolescents (age from 12.0 to 18.0). Accordingly, we analyzed data from 84 children with ADHD, 60 adolescents with ADHD, 84 children HC, and 72 adolescent HC. Their detailed clinical and demographic information is provided in Table 1.

**TABLE 1:** Demographic and clinical characteristics of subjects.

Characteristics	ADHD patients		Healthy controls		Comparison		
	Children (*n* = 84)	Adolescents (*n* = 60)	Children (*n* = 84)	Adolescents (*n* = 72)	ADHD vs. HC (144/156)	Children vs. adolescents (168/132)	All groups
	Mean ± SD	Mean ± SD	Mean ± SD	Mean ± SD			
Age	9.51 ± 1.32	14.16 ± 1.56	10.06 ± 1.56	13.94 ± 1.367	*p* = .106^a^	*p* < 0.0001^a^	*p* < 0.0001^b^
Sex (male/female)	54/30	48/12	56/28	44/28	*p* = .395^c^	*p* = .459^c^	*p* = .701^c^
FSIQ	106.0 ± 13.31	103 ± 13.80	116.6 ± 14.61	113.4 ± 12.5	*p* < .0001^a^	*p* = .310^a^	*p* < .0001^b^
VIQ	108.1 ± 13.23	106.7 ± 14.99	117.7 ± 14.29	113.6 ± 12.49	*p* < .0001^a^	*p* = .640^a^	*p* < .0001^b^
PIQ	102.2 ± 15.48	99.2 ± 14.07	113.3 ± 15.98	110.0 ± 13.2	*p* < .0001^a^	*p* = .1790^a^	*p* < .0001^b^
IA	65.01 ± 19.24	53.59 ± 21.59	31.54 ± 15.88	31.39 ± 16.89	*p* < .0001^a^	*p* = .024^a^	*p* < .0001^b^
HI	61.39 ± 21.99	48.78 ± 24.39	31.31 ± 17.09	30.61 ± 17.87	*p* < .0001^a^	*p* = .013^a^	*p* < .0001^b^

Abbreviations: FSIQ, Wechsler Abbreviated Scale of Intelligence (WASI) Full‐Scale IQ; HI, hyperactivity/impulsivity score; IA, inattention score; PIQ, performance IQ; VIQ, verbal IQ.

^a^ Two‐sample *t*‐test (using nonparametric test).

^b^ One‐way ANOVA (using nonparametric test).

^c^ Chi‐square test.

### 2.2 Image acquisition

The data used in this experiment were obtained by the NYU and PKU centers. At each site, the corresponding institutional review boards approved all procedures for data collection, and written informed consent was obtained from each participants' parents. For the NYU data, MRI data were obtained on a 3T Allegra MRI scanner at the NYU Center for Brain Imaging. The NYU scans were performed using a standard echo‐planar imaging pulse sequence with the following parameters: repetition time/echo time (TR/TE) = 2000 ms/15 ms, flip angle (FA) = 90°, field of view (FOV) = 240 mm, voxel size =3 × 3 × 4 mm^3^, number of slices = 33, slice thickness = 4 mm, 176 volumes. T1‐weighted anatomical data were acquired using an MPRAGE (MEMPR) sequence with scanning parameters as follows: TR/TE = 2530 ms/3.25 ms, FA = 7°, FOV = 256 mm, slice thickness = 1 mm, voxel size = 1.3 × 1 × 1.3 mm^3^, number of slices = 128, slice thickness = 1.33 mm. The anatomical data were used to normalize the functional images for each subject. MRI data from PKU were obtained on a 3T Trio MRI scanner. As parameters were set differently for each batch of PKU data, please refer to The Neuro Bureau ADHD‐200 Preprocessed repository for details (Bellec et al., 2017).

### 2.3 Functional magnetic resonance imaging (fMRI) data preprocessing

**FIGURE 1:**
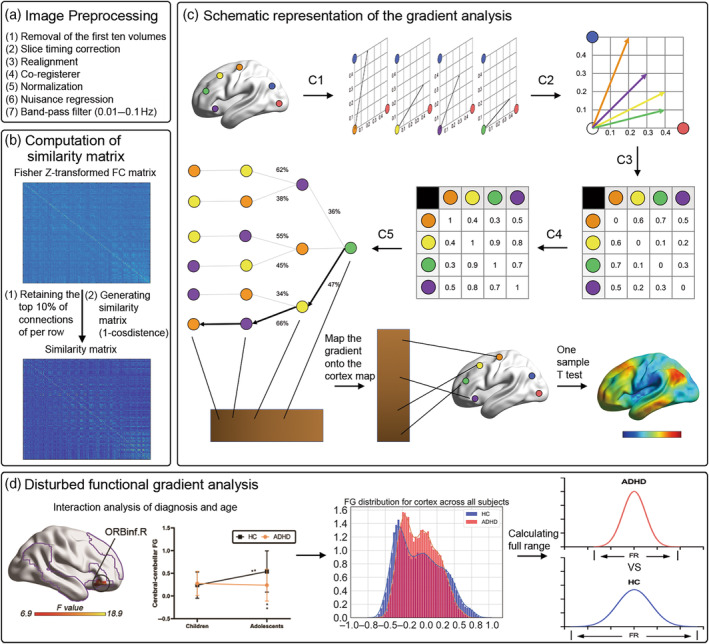
Study overview. (a) The steps for image preprocessing. (b) The steps to calculate the similarity matrix. (c) The details of calculating gradient matrix. C1–C3 represent the process of calculating the cosine similarity matrix and C4 shows the process of calculating the affinity matrix is (1‐cosine distance). A Markov chain is constructed using information from the affinity matrix. Information from the affinity matrix is thus used to represent the probability of transition between each pair of vectors (C5) A Markov chain is constructed using information from the affinity matrix. Information from the affinity matrix is thus used to represent the probability of transition between each pair of vectors. In this way, there will be higher transition probability between pairs of voxels with similar connectivity patterns. This probability of transition between each pair of vectors can be analyzed as a symmetric transformation matrix, thus allowing the calculation of eigenvectors. (d) The analysis procedure for the disturbed FG analysis in ADHD. ADHD, attention‐deficit/hyperactivity disorder; FG, functional gradient; FR, full range; HC, healthy controls; ORBinf, orbital part of inferior frontal gyrus.

All preprocessing was carried out using Data Processing & Analysis for Brain Imaging (DPABI) software (http://rfmri.org/dpabi) and MATLAB scripts (Figure 1a). The first step for functional images started with the removal of the first 10 volumes, slice‐timing, and head motion correction. Subsequently, the preprocessed images were co‐registered to the high‐resolution 3D anatomic volume. To standardize the brain registration of different shapes between subjects onto the same template to make it comparable, we used the MNI template during the preprocessing. All functional images in individual native space were transformed into Montreal Neurological Institute (MNI) with spatial resolution of 3 × 3 × 3 mm^3^. Nonrelevant signals were regressed out, including the linear trend, Friston 24 head motion parameters, and averaged white matter and cerebrospinal fluid signals (CompCor, 5 principal components; Behzadi et al., 2007). Temporal filtering was performed on the above preprocessed functional images in the low‐frequency range of 0.01–0.1 Hz to minimize the physiological noise.

### 2.4 Obtaining the cerebral networks and cerebellar mask

To associate the different clusters with unique brain networks, we used the seven functional networks defined by Yeo et al. (2011), that is, the default mode network (DMN), dorsal attention network, ventral attention network, FPN, limbic network, somatomotor network (SMN), and visual network (VN). The cerebellar mask was obtained from the MNI fluid‐attenuated inversion recovery Maxprobthr25‐2 mm mask available in FSLView Toolbox (http://www.fmrib.ox.ac.uk/fsl). Subjects with incompletely scanned cerebellums were excluded from further analysis.

### 2.5 Functional connectivity gradient analysis

Functional connectivity gradient analysis was performed using the BrainSpace toolbox (http://github.com/MICA-MNI/BrainSpace) and MATLAB scripts. Previous work has shown that diffusion map embedding identifies relevant aspects of functional organization in the human brain (Coifman et al., 2005;Guell et al., 2018; Margulies et al., 2016), resulting in multiple, continuous maps, namely gradients. Gradients represent the functional connectivity similarity of each voxel along a continuous space (Dong et al., 2020), which has proven useful for understanding the relationships between functions in specific regions and individual domains from sensation to cognition. This study analyzed cerebro‐cerebellar FG in children and adolescents with ADHD. We extracted voxel‐level functional signals within whole brain and performed Pearson's correlation analysis between cerebral cortex and cerebellum to create a functional connectivity matrix (44,123 × 5949) for each subject. That connectivity matrix was then Fisher's *Z* transformed and converted to a sparse matrix with 10% sparsity following previous work (Guell et al., 2018; Margulies et al., 2016). A cosine similarity matrix was calculated from the sparse matrix, reflecting the similarity of connectivity profiles between each pair of voxels (Figure 1b). Finally, diffusion map embedding was used to identify a low‐dimensional embedding from the high‐dimensional connectivity matrix (Coifman et al., 2005). In this algorithm, only one parameter *α* (ranging from 0 to 1) controls the sampling point density which affects the underlying manifold. When *α* = 0, the diffusion amounts to a normalized graph Laplacian with isotropic weights; for *α* = 1, it approximates the Laplace–Beltrami operator and when *α* = .5, it approximates Fokker–Planck diffusion (Coifman & Lafon, 2006). Since *α* is commonly set to .5 to retain global relations between data points in the embedded space, we chose *α* = .5 in line with previous studies (Margulies et al., 2016; Vos et al., 2018). BrainSpace toolbox provides three alignments, which are unaligned form, Procrustes alignment, and joint embedding. An increase in correspondence between individual subjects and the template manifold when Procrustes alignment was used compared with unaligned approaches, mainly driven by trivial changes in the sign of specific gradients in a subgroup of subjects. Joint embedding is an alternative to Procrustes alignment, and the gradients provided by this method can increase analysis across disciplines and species. A joint embedding generates a new manifold, which may lead to a new solution that may be slightly different from the initial gradient calculated separately. Considering the high computational cost of joint embedding, we finally chose to use Procrustes alignment (Hong et al., 2019; Langs et al., 2015). Figure 1c shows the specific process of gradient calculation. It illustrates a method to calculate the principle FGs of four cortical voxels (orange, yellow, green, purple) based on their functional connectivity with two target cortical voxels (blue, red). (C1) Functional connectivity from each cortical voxel (orange, yellow, green, purple) to two target cortical voxels (blue, red) are represented as two‐dimensional vectors. (C2) All vectors can be represented in the same two‐dimensional space. (C3) Calculating the cosine distance between each pair of vectors, and (C4) constructing the similarity matrix as (1‐cosine distance) for each pair of vectors. The similarity matrix represents the similarity of the connectivity patterns of each voxel pair. (C5) Constructing Markov chains using information from the similarity matrix. Thus, the information from the similarity matrix is used to represent the transition probability between each pair of vectors. In this way, there will be a higher transition probability between voxel pairs with similar connectivity patterns. This transition probability between each pair of vectors can be analyzed as a symmetric transformation matrix, allowing the eigenvector to be calculated. The eigenvectors derived from this transformation matrix represent the major orthogonal directions of the transformations between all pairwise voxels. Here, we illustrate the first result component of this analysis—based on the connectivity between the four brain voxels (orange, yellow, green, purple) and the two target cortical voxels (blue, red), whose principle FGs range from green to yellow, purple, orange voxels. This sequence is mapped back to our brain map, allowing us to generate functional neuroanatomical images. Notably, cortical FGs were calculated using the value of functional connections between each cortical voxel and the remaining cortical voxels (rather than just four voxels and two target voxels). Therefore, the vectors in our analysis are not limited to two dimensions, but can also calculate the cosine distance between pairs of higher dimensional vectors. Since the first gradient (i.e., the principal gradient) explains the maximum variance in the data (Dong et al., 2020), we used the first gradient for subsequent analyses. The same procedures were performed for cerebellar FG analyses. Finally, we mapped the gradient matrix to the cortical mask to obtain the corresponding gradient map for each subject.

We performed statistical analyses for all FG maps using SPM12 software (Figure 1d; http://www.fil.ion.ucl.ac.uk/spm/software/spm12). To explore within‐group gradient distributions for cortex and cerebellum, we performed one‐sample *t*‐tests on the FG maps for each group. Two‐way analysis of variance (ANOVA) was used to investigate the interaction effect between diagnosis (ADHD vs. HC) and age (children vs. adolescents) for cerebro‐cerebellar FG maps, controlling for sex and site. Gaussian random fields correction was performed on the above statistical maps (one‐tailed for F map: minimum *z* > 2.3; two‐tailed for T map: minimum *z* > 2.5 with cluster significance set to *p* < .05). Subsequently, post hoc analysis was performed to quantitatively measure the between‐group differences of cerebro‐cerebellar FG maps using the two‐sample *t*‐tests with Mann–Whitney *U* test.

### 2.6 Distribution analysis of FG maps

Since the interactions of diagnosis and age highlighted regions in the DMN and VN, histogram analysis was performed to systematically assess voxel‐level changes in FG distribution across DMN, VN, and cerebral cortex for ADHD and HC. Specifically, the corresponding shape and size of the histogram were used to display the FG distribution pattern within cortical voxels. Additionally, we performed the same analysis processes during the two developmental stages (children and adolescents separately) to assess whether diagnostic effects differed by age group.

Gradient distribution histogram analyses showed FG compression in the cerebral cortex. To quantitatively describe the compressed cerebro‐cerebellar FG in ADHD, we calculated the full range (FR) of principal gradient scores (Liang et al., 2021). The FR, reflecting the global distribution span of the principal gradient, was extracted between the largest positive scores and the smallest negative scores across all regions. Two‐sample tests with Mann–Whitney *U* tests were used to estimate whether the FR differed between ADHD and HC, controlling for sex and site.

### 2.7 Clinical correlation analysis

To assess relationships between cerebro‐cerebellar/cerebellar‐cortical FG differences and clinical symptom scores, we calculated their Pearson correlation coefficients. CPRS‐R‐LV (NYU center) or ADHD‐RS‐IV (PKU center) provided dimensional symptom measures of inattention and hyperactivity/impulsivity. Corresponding subscale scores for these two instruments demonstrate good convergent validity (Zhang et al., 2005). To control for differences in ranges of potential scores obtained from different instruments and to enable comparison across sites, symptom subscales were rescaled to have a range of 0.0–1.0 for each site by normalizing all scores to their corresponding maximum (Elton et al., 2014).

## 3 RESULTS

### 3.1 Experimental design

This study performed the gradient‐based functional connectivity analyses on resting‐state fMRI from publicly available ADHD‐200 dataset to investigate the disturbed cerebro‐cerebellar FG in children and adolescents with ADHD. The workflow of this study involved the following four steps (Figure 1): (A) Performing preprocessing analysis for all functional images. (B) Calculating the cerebro‐cerebellar connectivity matrix (44,123 × 5949) for each subject, and then generating a cosine similarity matrix. (C) Calculating gradient matrix and mapping onto the mask. (D) Investigating the interactions between diagnosis (ADHD vs. HC) and age (Children vs. Adolescents) in cerebro‐cerebellar FG, and then investigating the distributional differences in ADHD. The statistical analysis on clinical and demographic information was estimated in Table 1.

### 3.2 Within‐group cerebro‐cerebellar FG maps

**FIGURE 2:**
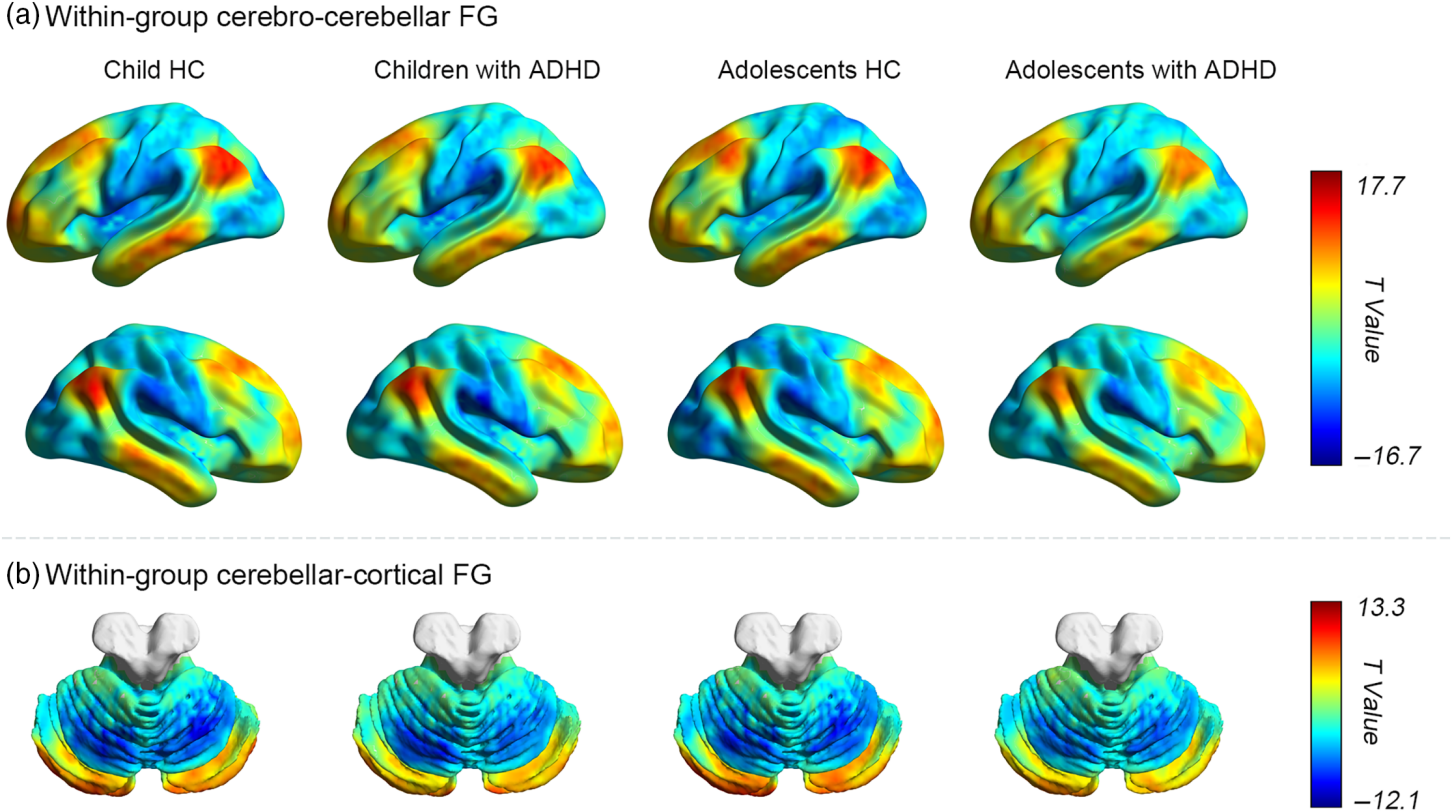
Group patterns in principal gradient. (a,b) The within‐group cortical and cerebellar functional gradient distribution patterns for four groups, respectively. Color bar shows *t* values. ADHD, attention‐deficit/hyperactivity disorder; FG, functional gradient; HC, healthy controls.

Within‐group results showed robust whole‐brain effects within each group (Figure 2). All four within‐group maps exhibited higher cerebro‐cerebellar FG in the DMN and lower cerebro‐cerebellar FG in sensorimotor and visual regions like the SMN and VN (Figure 2a). In addition, we also found similar FG distribution patterns in the cerebellum. Cerebellar posterior regions showed high FG values, while cerebellar anterior regions had lower FG values (Figure 2b).

### 3.3 FG analysis of cerebral cortex

The main effect of age showed increased cerebro‐cerebellar FG in superior frontal gyrus and left middle frontal gyrus in adolescents compared with children, and decreased FG in bilateral superior parietal gyrus, right superior temporal gyrus, and right middle occipital gyrus (Figure S1). We did not observe diagnosis main effect for cerebro‐cerebellar FG in cortex.

**FIGURE 3:**
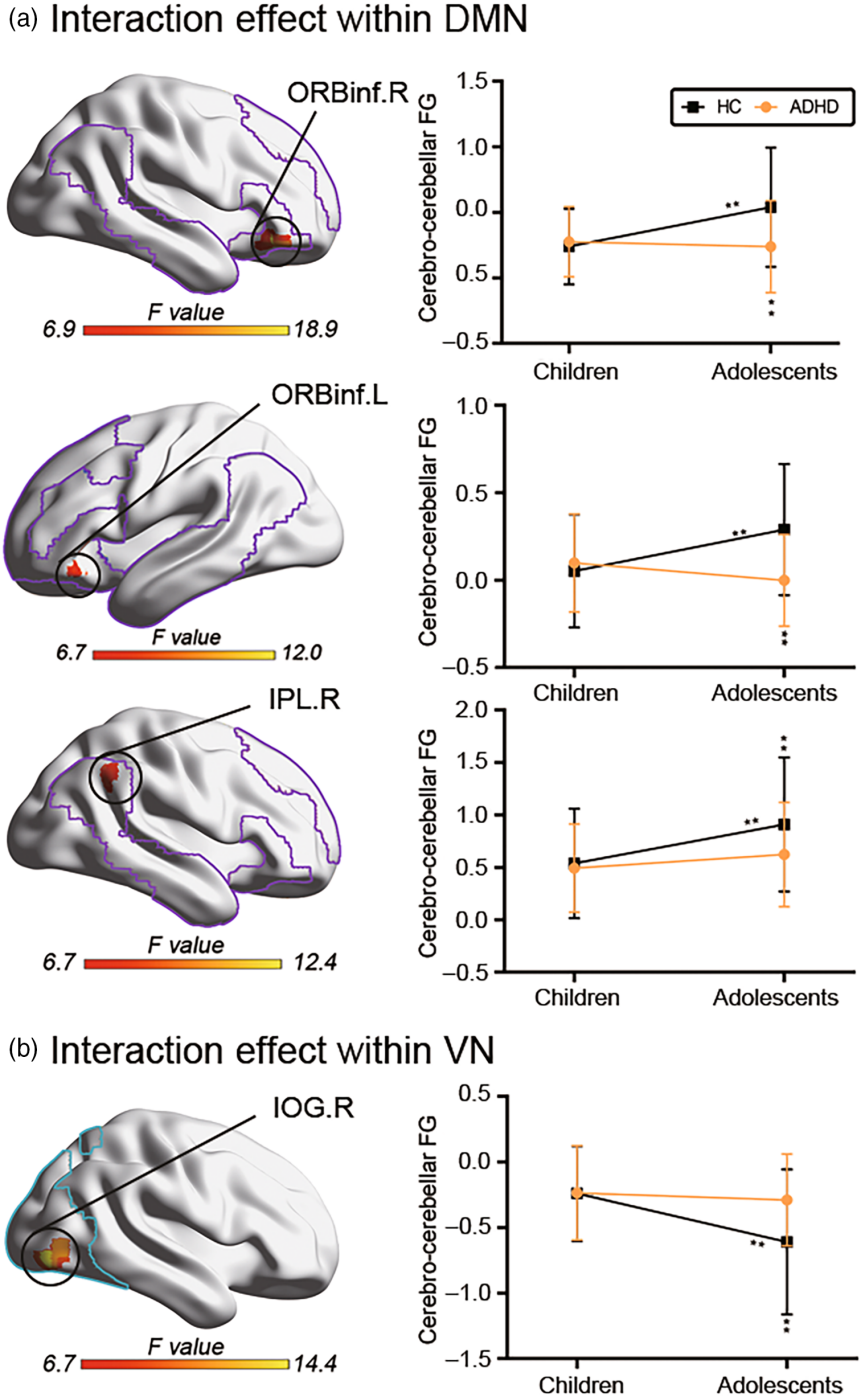
Interaction effect of cerebro‐cerebellar FG between diagnosis and age. (a) Left portion: Interaction in the bilateral ORBinf and right IPL within DMN. Right portion: Mean cerebro‐cerebellar gradient values and their standard deviation in these regions. (b) Left portion: Interaction in the right IOG within VN. Right portion: Mean cerebro‐cerebellar gradient values and their standard deviation. Statistical significance level was set at *p* < 0.05. Colored lines mapped on the cortical surface indicate the borders of DMN and VN. Note that statistical significance level was set at *p* < .05. **p < .05. ADHD, attention‐deficit/hyperactivity disorder; DMN, default mode network; FG, functional gradient; HC, healthy controls; IOG, inferior occipital gyrus; IPL, inferior parietal lobe; ORBinf, orbital part of inferior frontal gyrus; VN, visual network. The network maps are from Yeo 7 network.

Analysis of diagnosis and age group interaction revealed findings in the bilateral orbital part of inferior frontal gyrus (ORBinf) and right inferior parietal lobe (IPL) within the DMN (Figure 3a), and right inferior occipital gyrus (IOG) within the VN (Figure 3b). Specifically, adolescents with ADHD showed reduced cerebro‐cerebellar FG in the three above‐mentioned clusters within the DMN compared with adolescent HC (ORBinf.L: *u* = 1151, *p* < .0001; ORBinf.R: *u* = 1284, *p* < .0001; IPL.R: *u* = 1627, *p* = .0146). Adolescent HC exhibited increased cerebro‐cerebellar FG in these regions compared with child HC (ORBinf.L: *u* = 1936, *p* < .0001; ORBinf.R: *u* = 1830, *p* < .0001; IPL.R: *u* = 1981, *p* = .0002). There were no significant differences in FG between healthy and ADHD children in comparison of diseases, suggesting that the effects of disease on gradients are not significant in childhood. The FG of healthy adolescents was significantly higher than that of ADHD adolescents, indicating that the disease effect would cause a significant decrease in the gradient. In the comparison of age, the FG of healthy adolescents is significantly higher than that of healthy children, indicating that according to the normal development trajectory, the gradient value of healthy subjects will be significantly increased, that is, the influence of developmental effects on the gradient is to increase the gradient. There was no significant difference between the FG of ADHD children and ADHD adolescents, indicating that the intensity of the influence of developmental effects and disease effects on the gradient was similar, but the influence direction was opposite. Overall, the disease effect affected the normal developmental trajectory, so that the development of ADHD children to ADHD adolescents did not produce a significant increase in the gradient.

In contrast, the interaction effect involving IOG.R within the VN showed an opposite effect with increasing age. Specifically, adolescents with ADHD showed higher cerebro‐cerebellar FG in IOG.R compared with adolescent HC (*u* = 1384, *p* = .0003), and adolescent HC had lower FG in IOG.R compared with child HC (*u* = 1752, *p* < .0001). In the comparison of diseases, there was no significant difference in the FG between healthy children and ADHD children, which was the same as the results of DMN, indicating that the effect of disease on the gradient was not significant in the childhood stage. The FG of healthy adolescents was significantly lower than that of ADHD adolescents, indicating that the gradient was significantly increased by disease effects. In comparison of age, the FG of healthy adolescents is significantly lower than that of healthy children, indicating that according to the normal development trajectory, the gradient value of healthy subjects will be significantly reduced, that is, the influence of developmental effect on the gradient is to reduce the gradient. There was no significant difference between the FG of ADHD children and ADHD adolescents, indicating that the intensity of the influence of developmental effects and disease effects on the gradient was similar, but the influence direction was opposite. Overall, the disease effect affected the normal developmental trajectory, so that the development of ADHD children to ADHD adolescents did not produce a significant reduction in FG.

According to the results of DMN and VN, there was no significant difference between the gradient values of ADHD and HC in childhood, while the gradient values of HC would change significantly with the increase of age, while the gradient values of ADHD would not change significantly under the combined effect of developmental effects and disease effects. The developmental effect is promoting the change of the gradient value of the brain region, while the disease effect is suppressing this change, and the two effects are antagonistic.

### 3.4 FG analysis of cerebellum

**FIGURE 4:**
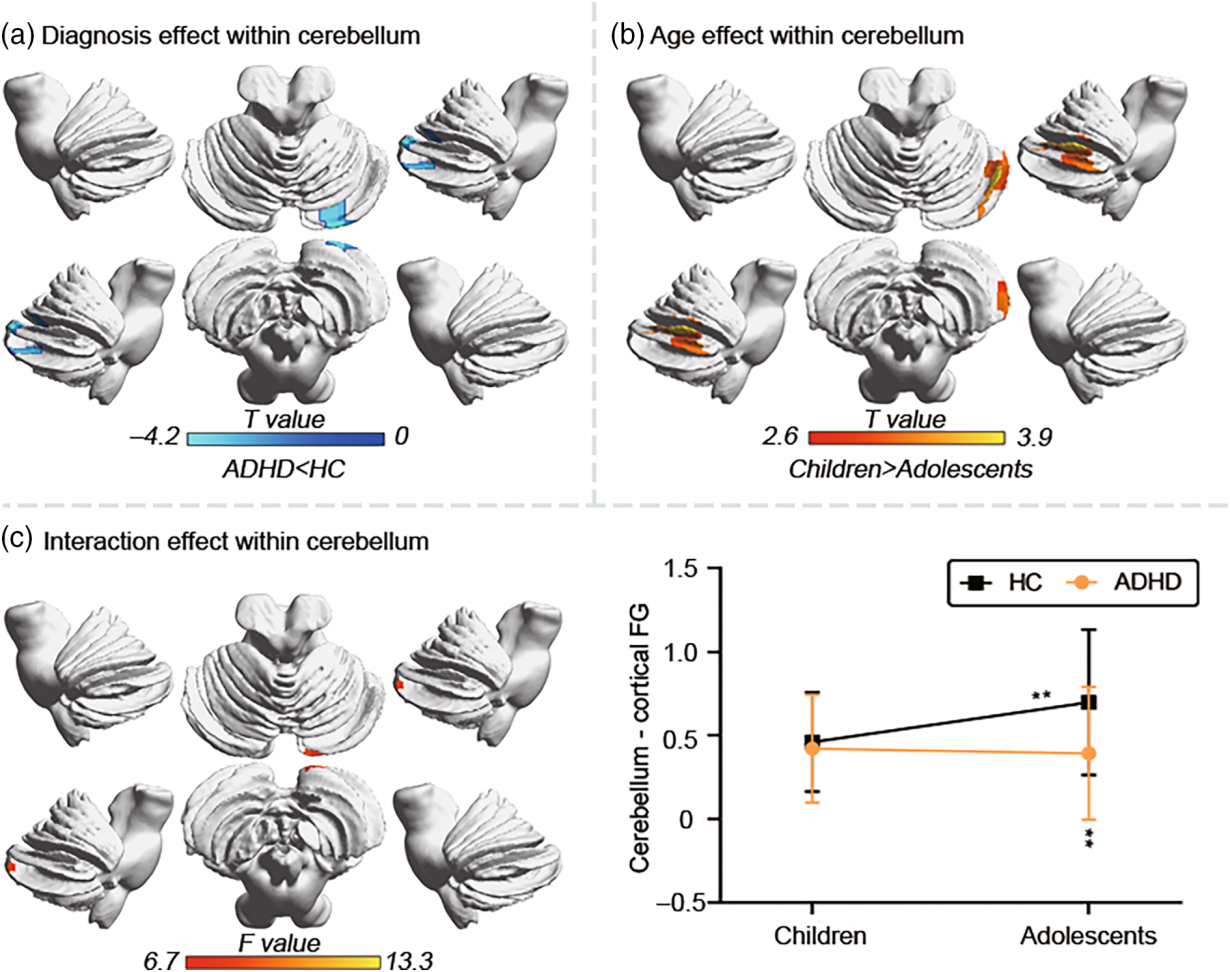
Interaction effect of cerebellar‐cortical FG between diagnosis and age. Two‐way ANOVA of cerebellar‐cortical gradient values shows the significant main effects of diagnosis (a) and age (b). (c) Left portion represents the regions of interaction effect distributing in the cerebellum_crus2. Right portion represents the averaged cerebellar‐cortical gradient values and their standard deviation. Note that statistical significance level was set at *p* < .05. **p < .05. ADHD, attention‐deficit/hyperactivity disorder; HC, healthy controls.

The main effect of diagnosis exhibited decreased cerebellar‐cortical FG in the cerebelum_crus1 in ADHD compared with HC (Figure 4a). The main effect of age showed decreased cerebellar‐cortical FG in the cerebelum_crus1 in adolescents compared with children (Figure 4b). Interaction analysis exhibited lower cerebellar‐cortical FG for the cerebellum_crus2 in adolescents with ADHD than in adolescent HC (*u* = 1291, *p* < .0001). Adolescent HC had higher cerebellar‐cortical FG for the cerebelum_crus2 than child HC (*u* = 2065, *p* = .0006; Figure 4c).

### 3.5 Compressed pattern of cerebro‐cerebellar FG

**FIGURE 5:**
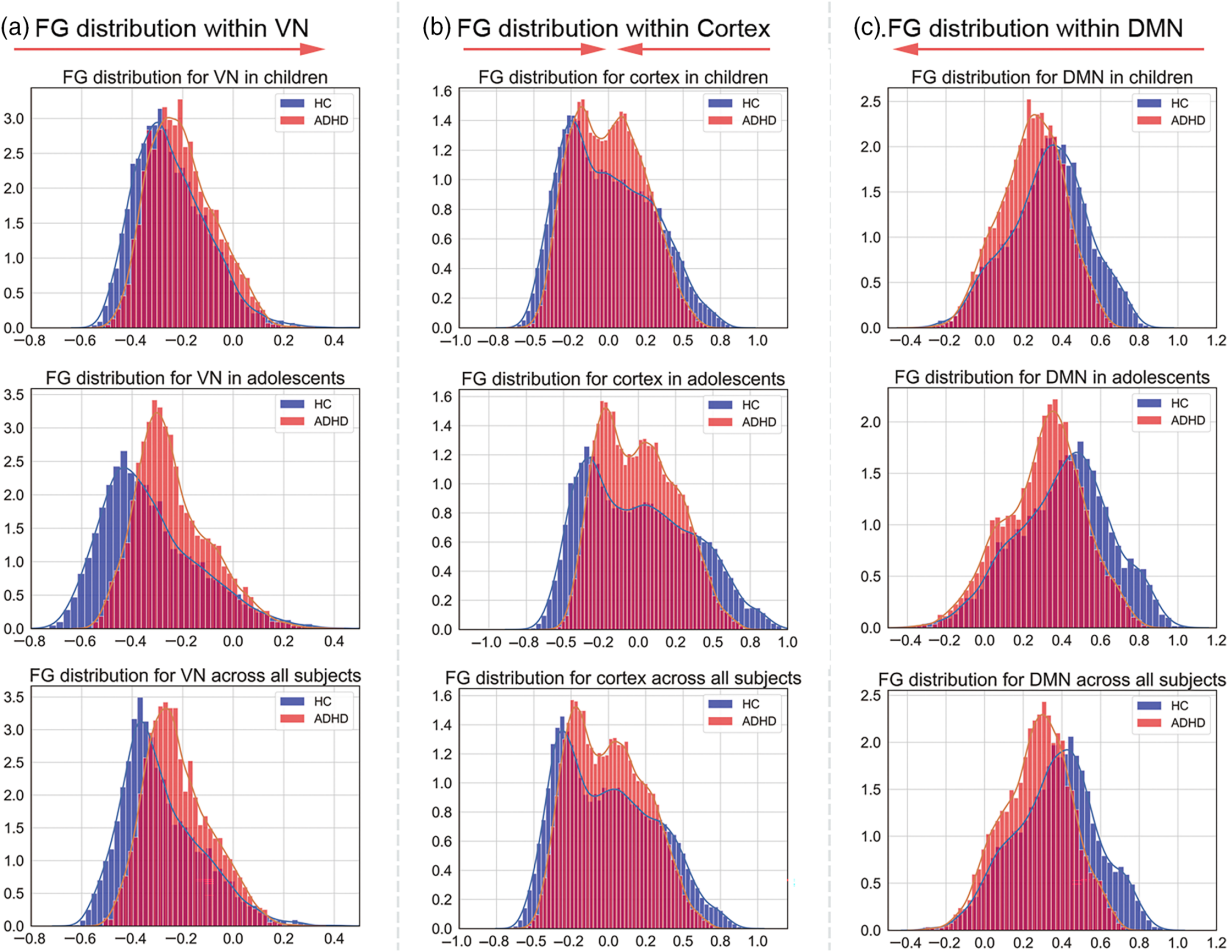
Compressed gradient pattern analysis shown in density histograms. (a,c) The FG distribution of ADHD and HC in VN and DMN, respectively. (b) represents the gradient distribution of ADHD and HC in cerebral cortex, which also represents the cortical‐cerebellar compressed gradient pattern. Each of A, B and C includes different developmental stages with children, adolescents and all subjects. Note that *x*‐axis: Gradient value; *y*‐axis: Quantity (that is, the number of voxels within a certain range). The curve in the figure has an area of 1 around the *x*‐axis. DMN, default mode network; FG, functional gradient; VN, visual network. The network maps are from Yeo 7 network.

**FIGURE 6:**
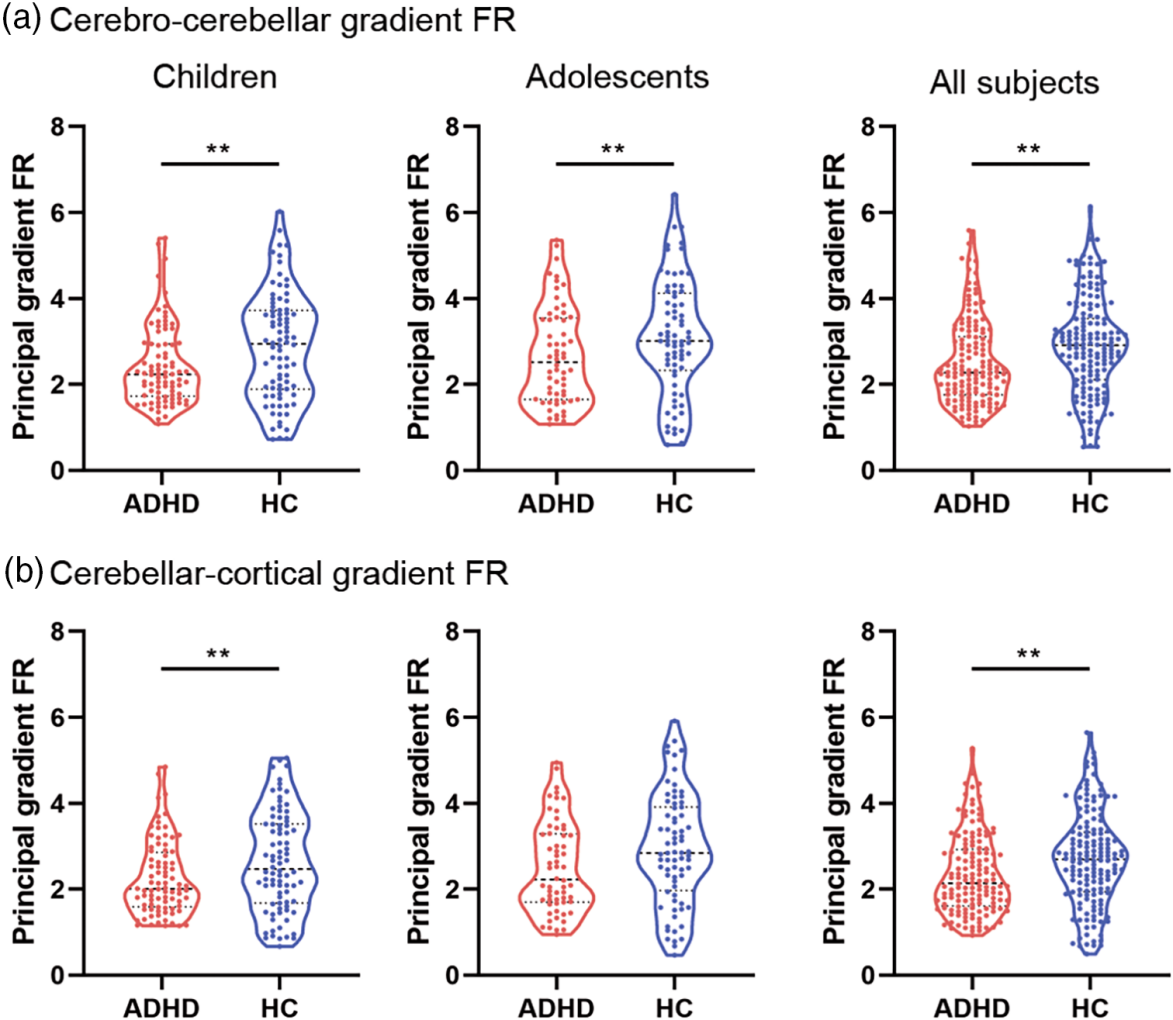
Group differences in range of the cerebro‐cerebellar and cerebellar‐cortical functional gradients. (a) Decreased range of cerebro‐cerebellum functional gradient in patients with ADHD in all developmental stages. (b) Decreased range of cerebellum‐cerebral cortical functional gradient in patients with ADHD in all developmental stages. Note that statistical significance level was set at *p* < .05. **p < .05. FR, full range.

Since the findings from the age by diagnosis interaction were mainly distributed in the DMN and VN, we performed histogram analyses on those networks as well as the entire cerebral cortex. Figure 5a shows that the cerebro‐cerebellar FG distribution pattern within the VN shifts to the right in ADHD compared with HC. The FG shift showed significant diagnostic differences both in children and adolescents (Figure S2A; Children: *u* = 12,911,152, *p* < .0001; Adolescents: *u* = 10,638,681, *p* < .0001; All subjects: *u* = 11,289,542, *p* < .0001). In contrast, Figure 5c shows the opposite FG shift within the DMN which was also significant for both developmental stages (Figure S2B; Children: *u* = 25,550,036, *p* < .0001; Adolescents: *u* = 26,313,392, *p* < .0001; All subjects: *u* = 26,388,400, *p* < .0001). Figure 5b shows the cerebro‐cerebellar FG distribution at the whole‐brain level with both sides shifting toward the center. Figure 6a shows the differences in FR between ADHD and HC. Regardless of age group, ADHD subjects showed significantly reduced cerebro‐cerebellar principal gradient FR (Children: *u* = 2735, *p* = .0117; Adolescents: *u* = 1731, *p* = .0499; All subjects: *u* = 8657, *p* = .0006), which quantitatively substantiates cerebro‐cerebellar FG compression in ADHD.We also estimated the cerebellar FG compressed pattern, and found that the entire cerebellum shifted toward the center (Figure S3). Figure 6b shows the cerebellar FR differences between ADHD and HC. Except for a trend for adolescents, participants with ADHD showed significantly reduced FR of cerebellar‐cortical FG in children and when both age ranges were combined (Children: *u* = 2822, *p* = .0250; Adolescents: *u* = 1739, *p* = .0544; All subjects: *u* = 8876, *p* = .0017), which quantitatively documented cerebellar‐cortical FG compression in ADHD.

### 3.6 Relationships between FG scores and clinical variables

**FIGURE 7:**
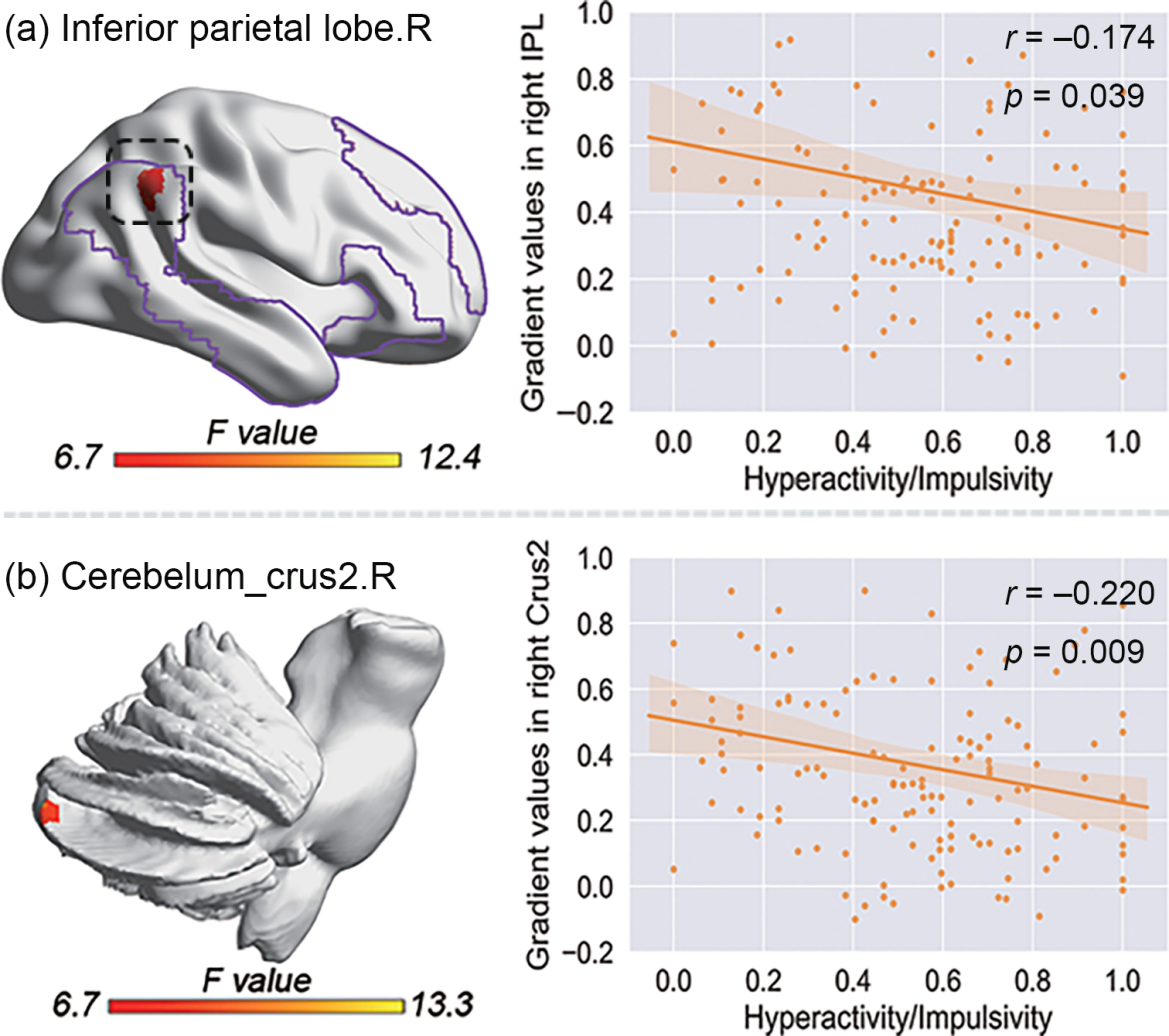
Relationships between cerebro‐cerebellar FG and clinical variables. (a) The negative correlations between cerebro‐cerebellar FG distributing in the IPL.R within DMN and hyperactivity/impulsivity. (b) The negative correlations between cerebellar‐cortical FG distributing in the cerebelum_crus2 and hyperactivity/impulsivity. Colored lines mapped on the cerebral surface indicate the borders of the DMN. Crus_2, cerebelum_crus2; DMN, default mode network; IPL, Inferior parietal lobe. The network maps are from Yeo 7 network. We found the abnormal interaction of diagnosis and age in DMN (default mode network) and VN (visual network) in ADHD. The opposite gradient change of VN portion and DMN portion leads to the compression of cortex in ADHD.

We found a negative correlation between cerebro‐cerebellar FG in the IPL.R (Figure 7a) within the DMN and a dimensional index of ADHD, that is, hyperactivity/impulsivity (*r* = −.174, *p* = .039). Additionally, we also found a negative correlation between cerebellar‐cortical FG in right cerebellum_crus2 and hyperactivity/impulsivity (Figure 7b) (*r* = −.220, *p* = .009). The correlation coefficients above have not been corrected with Bonferoni. Since we believed that even if the clinical correlation coefficients with *p*‐values were <.05 without correction, it has a significance, we reported above correlation results.

## 4 DISCUSSION

This study assessed interactions between diagnosis (ADHD vs. HC) and age (Children vs. Adolescents) and their differential diagnostic distributions by combining gradient‐based functional connectivity and histogram analysis methods. We found interactions between diagnosis and age in bilateral ORBinf and IPL.R within the DMN, and in IOG.R within the VN. The averaged FG values of IPL.R and cerebelum_crus2 were correlated with hyperactivity/impulsivity severity. Main effects of diagnosis revealed increased cerebellar‐cerebro FG in the cerebellum_crus1. FG distribution analysis revealed opposite FG shift in the DMN and VN in ADHD, corresponding to disrupted FG hierarchy in self‐related thought (DMN) and visual preprocessing (VN) functions. The opposite FG shift in DMN and VN results in the cerebral cortex FG compressed from both sides toward the center, and the compression pattern suggests that abnormal organization hierarchy in ADHD may reflect the combined effects of low‐ (visual processing) and high‐order (self‐related thought) dysfunction.

### 4.1 Cortical interaction effects between diagnosis and age

To explore whether the effects of ADHD interact with age in cerebro‐cerebellar FG, we divided each subject into either the child or adolescent group. Age‐diagnostic interactions derived in the study were mainly distributed in the DMN and VN. DMN in adolescents with ADHD is associated with severity of clinical and somatic symptoms of ADHD (Kim et al., 2022). Additionally, although ADHD is a behavioral heterogeneous disorder, visual motor deficits have been found to be one of the common clinical symptoms of ADHD in a large number of studies (Carames et al., 2022; Fabio et al., 2022; Sani et al., 2022). In terms of brain regions, we observed significant interactions distributed in bilateral ORBinf and IPL.R within the DMN (Figure 3a) and IOG.R within the VN (Figure 3b) across all the subjects. Specifically, when contrasting children to adolescents, participants with ADHD exhibited reduced cerebro‐cerebellar FG in bilateral ORBinf and IPL.R within the DMN compared with HC. Additionally, adolescent HC showed significantly increased cerebro‐cerebellar FG in those regions compared with child HC. Per the normal developmental trajectory, FG values for bilateral ORBinf and IPL.R should increase significantly from childhood to adolescence. However, we observed that ADHD weakened the influence of development on cerebro‐cerebellar connections in these regions, resulting in significant differences between ADHD and HC adolescents. We note that ORBinf is located in the orbitofrontal cortex which is involved in reward (de Leeuw et al., 2015), controlling impulsive behaviors (Berlin et al., 2004) and risky decision‐making (Stalnaker et al., 2015), and IPL plays important roles in integrating visual, auditory and somatosensory information to guide movement (Caspers et al., 2011; Sandström et al., 2022). Previous studies have reported strong links between these two regions and impulsive behavior/inattention in ADHD (Cyders et al., 2015). Accordingly, we speculate that decreased cerebro‐cerebellar FG in bilateral ORBinf and IPL.R is involved in the impulsive behavior and inattention symptoms of ADHD adolescents, and this may underlie increasing conflicts with parents and development of anxiety disorders and even substance abuse in adolescents with ADHD (Biederman et al., 1998). In addition, since the bilateral ORBinf and IPL.R regions are located in the DMN, which is related to self‐related thoughts (Rohr et al., 2017), our findings also suggest abnormal information integration within DMN in ADHD, which might be affected by cerebellar dysregulation.

Additionally, we observed interactions between diagnosis and age in the IOG.R within the VN (Figure 3b). According to the normal development trajectory, cerebro‐cerebellar FG in IOG.R should decrease significantly as children develop to adolescents, but ADHD weakened the influence of development, suppressing significant changes, resulting in adolescents with ADHD having significantly higher cerebro‐cerebellar FG in IOG.R compared with adolescent HC. The IOG is located in the occipital lobe within the visual cortex and the primary visual area which is associated with visual processing (de Hass et al., 2021). Abnormal IOG.R FG might relate to visual‐motor deficits detected in children with ADHD ages 9–11 (Fabio et al., 2022).

### 4.2 Cerebellar interaction effects between diagnosis and age

The main effect of diagnosis in the cerebellum occurred in the right cerebelum_crus1 (Figure 4a). Compared with HC, the FG in this region was lower in ADHD. The main effect of age also occurred in the right cerebellum_crus1 (Figure 3b). Compared with adolescents, FG values were higher in children. An interaction between diagnosis and age was found in the right cerebellum_crus2 (Figure 4c). Per the normal developmental trajectory, the cerebellum becomes more differentiated (Gilmore et al., 2018) and the principal axis of cerebellar macroscale functional organization expands. Thus, the FG value in the cerebelum_crus2 increased significantly from HC children to adolescents, but in the ADHD groups the developmental effect was nullified, leading to the significant decrease of cerebelum_crus2 FG in adolescents with ADHD compared with adolescent HC. The cerebelum_crus1 and cerebelum_crus2 belong to the supramodal cognition regions of the cerebellum (Stoodley, 2012). A recent study reported significant activation in cerebellar cru1 and crus2 during anger and threat processing tasks (Klaus & Schutter, 2021). Similarly, emotional impulsivity, irritability, and increased aggression are also pathological features of patients with ADHD (Barkley & Fischer, 2010; Nigg, Karalunas, Gustafsson, et al., 2020). Combined with these studies, we speculate that inattention, impulsivity, and hyperactivity in ADHD are associated with structural and functional abnormalities of cerebellar crus1 and 2.

### 4.3 Compressed pattern of cerebro‐cerebellar FG

In this study, we observed compressed patterns of cerebro‐cerebellar FG. We found cerebro‐cerebellar FG shifting to the right within the VN (Figure 5a), reflecting higher FG values in ADHD compared with HC (Figure S2A). In contrast, we found cerebro‐cerebellar FG shifting to the left within the DMN (Figure 5c), reflecting lower FG values in ADHD compared with HC (Figure S2B). We also observed the cerebro‐cerebellar FG compression of the both sides towards center within the whole cerebral cortex (Figure 5b). Cortical FG extended from primary cortices like visual regions to the DMN (Margulies et al., 2016), suggesting opposite changes in FG values within DMN and VN are abnormalities associated with ADHD. This reflects compression of the lowest and highest portions of the principal axis in cerebral macroscale organization. From the perspective of brain anatomy perspective, “both sides shifted to the center” indicates that the brain regions located in the primary network (gradient value <0, on the left side of the histogram) and the brain regions located in the supramodal network (gradient value >0, on the right side of the histogram) have less difference in functional hierarchy and lower differentiation of the whole brain. The greater the difference in the gradient values of the two regions, the greater the functional difference between the two regions. Therefore, the compressed shorter gradient axis in ADHD indicates that the differences in the various brain regions located on the axis are reduced. The gradient value of a certain region is not meaningful in isolation, it is necessary to be combined with the global gradient axis (i.e., the principal gradient axis mentioned in this study). The position of its gradient value on the gradient axis represents its position in the global functional hierarchy, that is, its function. The greater differences of gradient values between two regions, the greater functional differences, and vice versa (Dong et al., 2020). The change of gradient values means that its position in the global functional hierarchy has changed, which will cause functional disorders. For instance, a brain region with a change in gradient value may be over‐involved in tasks in other brain regions and under‐involved in the original function, so that the state of “Each performs one's own functions” is broken (Hong et al., 2019). Our results show that the gradient value of DMN decreases, so the functional hierarchy difference between DMN and the lower‐order network will be reduced, and the lower‐order network may be over‐involved in the higher‐order tasks of DMN. For example, when learning or thinking, ADHD patients have symptoms such as involuntary hand and foot twitching, wriggling in the seat, suggesting that the sensorimotor network may be overly involved in higher‐order network tasks. A recent systematic analysis documented oculomotor deficits in ADHD and noted substantial overlap between the VN, involved in oculomotor control, and high‐order cognitive control processes involving the DMN, such as attention, planning, and inhibition (Maron et al., 2021). Thus the cortical FG compression pattern we observed might be associated with the pathological mechanisms underlying oculomotor deficits in ADHD. Additionally, a similar compressed gradient pattern was observed in the cerebellum (Figure S3) Therefore, we hypothesized that the gradient changes in the cerebellum of ADHD patients also occurred in the functional subregions responsible for self‐related thought and visual preprocessing.

Brain networks exhibit the property of hierarchical modularity, facilitating the separation of different functional regions. This feature also enables specific information transfer within and between modules for more complex and integrated mental activity (Meunier et al., 2010). A compressed architecture shows less hierarchical difference between the low‐order visual region and high‐order cognitive regions like the DMN (Murphy et al., 2018). The compressed pattern in ADHD indicates a breakdown of the inherent separation between the DMN and VN, and a decrease in differentiation of this global hierarchy might be a relevant feature of macroscale functional organization in ADHD. These findings suggest that abnormal hierarchical organization may be the basis of ADHD pathology and emphasize the relevance of the cerebellum in such abnormal macroscopic organization.

Moreover, cerebellum and cortex share a similar hierarchical organization, gradually progressing from unimodal to supramodal streams of information processing (Guell et al., 2018), suggesting that the pattern extending from primary regions to DMN also applies to intracerebellum connectivity. This study also found a consistent compression pattern between cerebro‐cerebellar and cerebellar‐cortical FG, further confirming the similar hierarchical organization in cerebellum and cortex (Guell et al., 2018). This suggests that study of the cerebellum may be of value in understanding pathological mechanisms underlying ADHD, especially from the perspective of changes across developmental stages.

### 4.4 Relationships between FG and clinical variables

We characterized relationships between abnormal cerebro‐cerebellar/cerebellar‐cortical FG clusters and clinical manifestations, and found that cerebro‐cerebellar FG values in IPL.R within the DMN were negatively associated with hyperactivity/impulsivity (Figure 7a). Similarly, we found negative correlations between cerebellar‐cortical FG in the cerebelum_crus2.R and hyperactivity/impulsivity (Figure 7b), confirming that the alterations of cerebro‐cerebellar/cerebellar‐cortical FG within DMN are relevant for understanding the substrates of hyperactive/impulsive behavior in ADHD (Cyders et al., 2015).

Although our findings are promising, limitations need to be considered. First, our data were exclusively cross‐sectional. Since cerebral/cerebellar neuroimaging changes differ across developmental stages, the lack of longitudinal data does not allow us to clearly elucidate FG changes in cerebral/cerebellar developmental changes between the two. Longitudinal data are needed for future research to definitively address FG changes in cerebral/cerebellar development. Also, the analysis by its very nature could only imply correlation between the two measures and not causality. Further analysis with longitudinal data can help answer these questions. Second, medication status data were unavailable for these subjects. Since methylphenidate has been shown to affect the resting state functional connectivity of cerebellum (Yoo et al., 2018), medication status data are needed to evaluate the potential effect of medication on current findings. Third, we observed that compared with the controls, individuals with ADHD exhibited the significantly lower IQ scores. This might be due to the impairment of cognitive function caused by the disease, which in turn affects IQ. However, this study focused on the cortical‐cerebellar FG, and it was difficult for us to investigate its specific reasons. Future research would be necessary to answer this important question. Fourth, we lacked an independent verification set to validate the findings of this study. But given that ADHD is a highly heterogeneous disorder (Feczko & Fair, 2020; Karalunas & Nigg, 2020; Nigg, Karalunas, Feczko, & Fair, 2020), too much pursuit of consistent outcomes can lead to an incomplete understanding of the disorder. Therefore, the focus of this study is to explore the functional level abnormalities of ADHD and the corresponding clinical significance. Indeed as more independent data becomes available, data analytics methods in addition to correlation‐based methods can be used to identify other measures of outcomes including FG between diagnosis and age. Finally, although we found a correlation between FG and clinical variables of ADHD, we were unable to establish causal relationships between changes in FG and the pathogenesis of ADHD. Future studies incorporating pre‐ and postintervention imaging are needed to explore the causal relationships between altered cerebellar gradients and neuropsychiatric behavior.

## 5 CONCLUSION

This study demonstrated the interactions between diagnosis and age in the bilateral ORBinf and IPL.R within the DMN, and IOG.R within the VN, which were correlated to ratings of hyperactivity/impulsivity in ADHD. Examination of FG compression revealed that the cerebro‐cerebellar FG compression from both sides to the center is due to the opposite FG shifts within DMN and VN, indicating their distinct contributions to disturbances in cerebral and cerebellar organizational hierarchy in ADHD.

## FUNDING INFORMATION

This study is supported by the Sichuan Science and Technology Program (grant number: 2024NSFSC1661), National Natural Science Foundation of China (grant number: 62171101, 82250410380), and China MOST2030 Brain (Project No. 2022ZD0208500).

## CONFLICT OF INTEREST STATEMENT

The authors declare no conflicts of interest.

## Supporting information


**Data S1:** Supporting information.

## Data Availability

The data that support the findings of this study are available from the corresponding author upon reasonable request.
