# Supplementary material for: Compressed cerebro‐cerebellar functional gradients in children and adolescents with attention‐deficit/hyperactivity disorder
Source: Hum Brain Mapp. 2024 Sep 10;45(13):e26796. doi: 10.1002/hbm.26796 (PMC11386319; doi:10.1002/hbm.26796)
Supplement: Supplementary file 1 — Data S1: Supporting information. [file HBM-45-e26796-s001.docx]

**Supplementary material 1**

This part shows the age main effect of the cerebro-cerebellar functional gradient.

We observed that children exhibited lower cerebro-cerebellar gradient values in the right medial superior frontal gyrus and left middle frontal gyrus than those of adolescents. Additionally, children exhibited higher cerebro-cerebellar gradient values in the bilateral superior parietal gyrus, right superior temporal gyrus and right middle occipital gyrus than those of Adolescents.


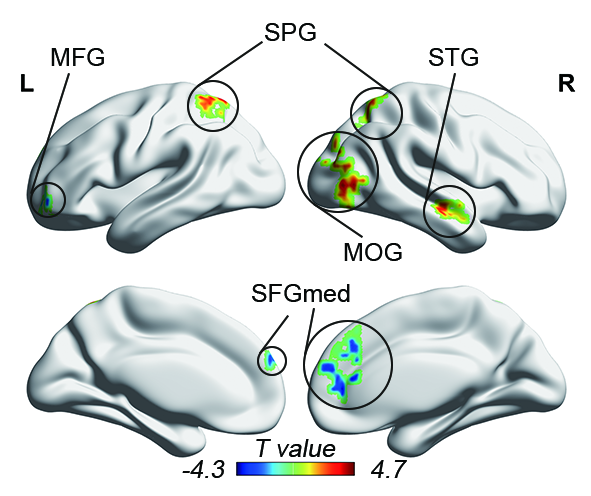


**Figure S1.** **The age main effect of cerebro-cerebellar functional gradient.** SFGmed = Medial superior frontal gyrus; MFG = Middle frontal gyrus; SPG = Superior parietal gyrus; STG = Superior temporal gyrus; MOG = Middle occipital gyrus.

**Supplementary material 2**

This part shows the gradient compression pattern analysis within cortex and cerebellum in ADHD.

We used the two-sample t tests to analyze the differences of functional gradient values within DMN and VN in ADHD compared to HCs. Figure S2A showed that functional gradient values distributing in VN are higher in ADHD compared to HCs at any development stage (Children: p < 0.0001, u = 25550036; Adolescents: p < 0.0001, u = 26313392; All Subjects: p < 0.0001, u = 26388400). Figure S2B shows that functional gradient values distributing in DMN were lower in ADHD compared to HCs at any development stage (Children: p < 0.0001, u = 12911152; Adolescents: p < 0.0001, u = 10638681; All Subjects: p < 0.0001, u = 11289542).

Then we calculated the average gradient values of each voxel in the cerebellum for all subjects across both developmental stages (Figure S3). We found a compression of the lowest portion and the highest portion of the principal axis of cerebellar macroscale organization.


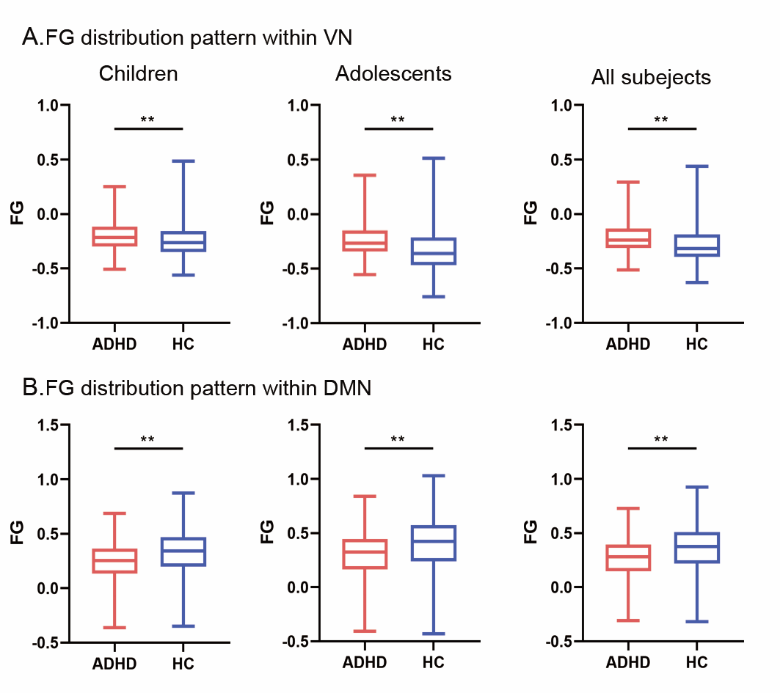


**Figure S2. Group differences in range of the cerebro–cerebellar functional gradient within DMN and VN.** (A) Increased functional gradient within VN in ADHD at all developmental stages. (B) Decreased functional gradient within DMN in ADHD at all developmental stages. Statistical significance level was set at p < .05. **p < .05.

**
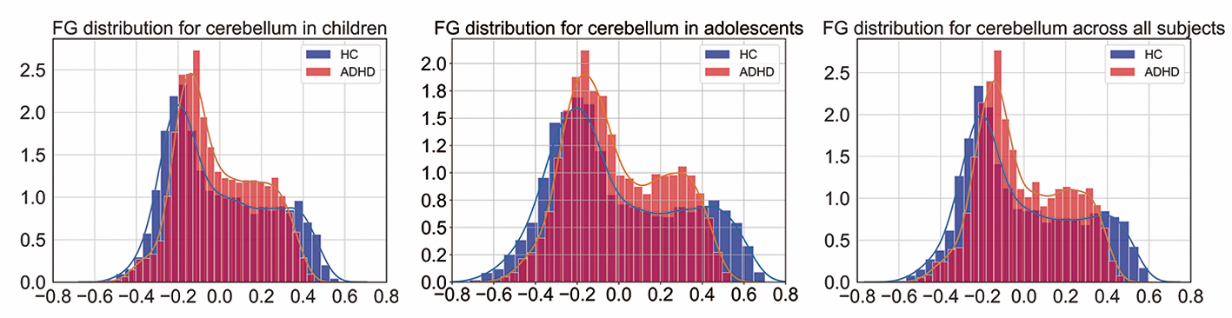
Figure S3. Compressed gradient pattern of cerebellar-cortical functional gradient** **at different development stage.** From left to right are the functional gradient density histograms of children, adolescents and all subjects.
